# Nationwide Turkish Cohort Study of Hypophosphatemic Rickets

**DOI:** 10.4274/jcrpe.galenos.2019.2019.0098

**Published:** 2020-06-03

**Authors:** Zeynep Şıklar, Serap Turan, Abdullah Bereket, Firdevs Baş, Tülay Güran, Azad Akberzade, Ayhan Abacı, Korcan Demir, Ece Böber, Mehmet Nuri Özbek, Cengiz Kara, Şükran Poyrazoğlu, Murat Aydın, Aslı Kardelen, Ömer Tarım, Erdal Eren, Nihal Hatipoğlu, Muammer Büyükinan, Nesibe Akyürek, Semra Çetinkaya, Elvan Bayramoğlu, Beray Selver Eklioğlu, Ahmet Uçaktürk, Saygın Abalı, Damla Gökşen, Yılmaz Kor, Edip Ünal, İhsan Esen, Ruken Yıldırım, Onur Akın, Atilla Çayır, Emine Dilek, Birgül Kırel, Ahmet Anık, Gönül Çatlı, Merih Berberoğlu

**Affiliations:** 1 Ankara University Faculty of Medicine, Department of Pediatric Endocrinology, Ankara, Turkey; 2 Marmara University Pendik Training and Reseach Hospital, Clinic of Pediatric Endocrinology, İstanbul, Turkey; 3 İstanbul University Faculty of Medicine, Department of Pediatric Endocrinology, İstanbul, Turkey; 4 Dokuz Eylül University Faculty of Medicine, Department of Pediatric Endocrinology, İzmir, Turkey; 5 University of Health Sciences Turkey, Gazi Yaşargil Training and Research Hospital, Clinic of Pediatric Endocrinology, Diyarbakır, Turkey; 6 Ondokuz Mayıs University Faculty of Medicine, Department of Pediatric Endocrinology, Samsun, Turkey; 7 Uludağ University Faculty of Medicine, Department of Pediatric Endocrinology, Bursa, Turkey; 8 Erciyes University Faculty of Medicine, Department of Pediatric Endocrinology, Kayseri, Turkey; 9 Konya Training and Research Hospital, Clinic of Pediatric Endocrinology, Konya, Turkey; 10 University of Health Sciences Turkey, Ankara Dr. Sami Ulus Obstetrics and Pediatrics Training and Research Hospital, Clinic of Pediatric Endocrinology, Ankara, Turkey; 11 Necmettin Erbakan University, Meram Faculty of Medicine, Department of Pediatric Endocrinology, Konya, Turkey; 12 Ankara City Hospital, Children’s Hospital, Clinic of Pediatric Endocrinology, Ankara, Turkey; 13 İstanbul Kartal Dr. Lütfi Kırdar Training and Research Hospital, Clinic of Pediatric Endocrinology, İstanbul, Turkey; 14 Ege University Faculty of Medicine, Department of Pediatric Endocrinology, İzmir, Turkey; 15 University of Health Sciences Turkey, Adana Numune Training and Research Hospital, Clinic of Pediatric Endocrinology, Adana, Turkey; 16 Dicle University Faculty of Medicine, Department of Pediatric Endocrinology, Diyarbakır, Turkey; 17 Fırat University Faculty of Medicine, Department of Pediatric Endocrinology, Elazığ, Turkey; 18 Diyarbakır Children Hospital, Clinic of Pediatric Endocrinology, Diyarbakır, Turkey; 19 University of Health Sciences Turkey, Gülhane Training and Research Hospital, Clinic of Pediatric Endocrinology, Ankara, Turkey; 20 Erzurum Training and Research Hospital, Clinic of Pediatric Endocrinology, Erzurum, Turkey; 21 Trakya University Faculty of Medicine, Department of Pediatric Endocrinology, Edirne, Turkey; 22 Eskişehir Osmangazi University Faculty of Medicine, Department of Pediatric Endocrinology, Eskişehir, Turkey; 23 Aydın Adnan Menderes University Faculty of Medicine, Department of Pediatric Endocrinology, Aydın, Turkey; 24 İzmir Katip Çelebi Faculty of Medicine, Department of Pediatric Endocrinology, İzmir, Turkey

**Keywords:** Hypophosphatemic rickets, PHEX, treatment

## Abstract

**Objective:**

Hypophosphatemic rickets (HR) is a rare renal phosphate-wasting disorder, which is usually X-linked and is commonly caused by PHEX mutations. The treatment and follow-up of HR is challenging due to imperfect treatment options.

**Methods:**

Here we present nationwide initial and follow-up data on HR.

**Results:**

From 24 centers, 166 patients were included in the study. Genetic analysis (n=75) showed *PHEX* mutation in 80% of patients. The mean follow-up period was 6.7±2.4 years. During the first 3-years of treatment (n=91), mild increase in phosphate, decrease in alkaline phosphatase and elevation in parathyroid hormone (PTH) levels were detected. The height standard deviation scores were -2.38, -2.77, -2.72, -2.47 at initial, 1^st^, 2^nd^ and 3^rd^ year of treatment, respectively (p>0.05). On follow-up 36% of the patients showed complete or significant improvement in leg deformities and these patients had similar phosphate levels at presentation with better levels in 1^st^ and 2^nd^ years of treatment; even the treatment doses of phosphate were similar. Furthermore, 27 patients developed nephrocalcinosis (NC), the patients showed no difference in biochemical differences at presentation and follow-up, but 3^rd^ year PTH was higher. However, higher treatment doses of phosphate and calcitriol were found in the NC group.

**Conclusion:**

HR treatment and follow-up is challenging and our results showed higher treatment doses were associated with NC without any change in serum phosphate levels, suggesting that giving higher doses led to increased phosphaturia, probably through stimulation of fibroblast growth factor 23. However, higher calcitriol doses could improve bone deformities. Safer and more efficacious therapies are needed.

**What is already known on this topic?**

Hypophosphatemic rickets (HR) is a rare renal phosphate wasting disorder commonly with X-linked inheritance. There is no nationwide data on HR with initial and follow-up findings.

**What this study adds?**

The age of diagnosis was similar in good and bad responders to conventional therapy. Good responders had better height standard deviation score on admission. Higher treatment doses led to nephrocalcinosis without any change in serum levels of phosphorus. Awareness of the importance of early diagnosis and treatment complications should be improved.

## Introduction

Hypophosphatemic rickets (HR) is a rare renal phosphate-wasting disorder caused by several genetic mutations in factors leading to increase in fibroblast growth factor 23 (FGF23) signalling or secretion, and in renal phosphate transporters (1). The most common inherited form of HR is X-linked HR (XLH; OMIM: #307800), where inactivating mutations of the Phosphate Regulating Endopeptidase Homolog, X-Linked (*PHEX*, OMIM: #300550) gene lead to local and systemic effects (2). The incidence of XLH is 1/20 000 live births, and it accounts for approximately 80% of familial cases (3).

PHEX is predominantly expressed in osteoblasts and down-regulation of PHEX increases serum levels of the phosphatonin, FGF23. FGF23 has a central role in HR pathophysiology. Elevated levels of serum FGF23 increase urinary phosphate excretion by downregulating renal sodium-phosphate transporters, and decrease intestinal phosphate absorption by restricting active vitamin D synthesis (2). The other rare genetic disorders of excess FGF23 are autosomal dominant HR (caused by missense mutation in *FGF23*), autosomal recessive HR (type 1 caused by mutations in the gene encoding dentin matrix protein *(DMP1)*, type 2 caused by mutations in ectonucleotide pyrophosphate/phosphodiesterase 1 *(ENPP1)* and type 3 caused by mutation in the family with sequence similarity 20, member C *(FAM20C)* gene (4).

Clinical presentation of HR includes rickets, osteomalacia, short stature, leg deformities, dental abscesses, premature cranial synostosis, and muscle weakness in children, and also pseudofractures, osteoarthritis, and entesopathy in adults. The phenotype can vary widely, even in the same family and diagnosis can be delayed (5). In addition to the rarity and diagnostic difficulties, which has a significant impact on patient outcomes, treatment and follow-up of HR is very challenging.

Conventional treatment of HR includes a combination of oral phosphorus and active vitamin D. Unfortunately this therapy was unsuccessful in a significant proportion of patients in respect to healing of rickets and improvement in deformities, and can be associated with treatment related side effects (4,6).

Information about the clinical, laboratory, genetic and follow-up characteristics of HR patients is very scarce for our population and only a few small series have been reported (7,8).

The aim was to present nationwide data on HR with initial and follow-up data on the patients presenting to pediatric endocrinology clinics before the age of 18 years.

## Methods

In this study, the data of 166 children and adolescents with HR who were being followed in 24 centers in Turkey were cross-sectionally analyzed . A nation-wide web-based CEDD-NET Data System (http://cedd.saglik-network.org/) was used for data collection between December 2016 and April 2018. A proforma, including clinical, genetic, laboratory and follow-up information about the patients was uploaded to the website and filled by the patient’s doctors. Study approval was given by the Ankara University Ethics Committee (approval number: 06-229-16).

Patients aged between 0 to18 years were included in the study and patients with calciopenic rickets (related to vitamin D deficiency or hypocalcemia, vitamin D dependent rickets, and the like) were excluded.

The following data on the patients’ admission, clinical and laboratory characteristics were collected: birth weight, age at diagnosis, age of first symptoms, positive family history, the time of starting to walk, height, weight, height standard deviation (SD) score (SDS), limb deformities (genu varum, genu valgum, etc.) with intercondylar and intermalleolar distance, other skeletal deformities (cranial, thoracal) and craniosynostosis, dental abscess, serum calcium (Ca), phosphorus, alkaline phosphatase (ALP), parathyroid hormone (PTH), 25-hydroxyvitamin D (25-OHD3) levels, tubular phosphate reabsorption (TPR), urinary Ca/creatinine ratio, and radiological findings. The researchers were also asked to enter other clinical features that were not included on the questionnaire form to the system. ALP SD of patients were calculated according to reference data (9).

The questionnaire form also included the genetic test results of the patient. Information concerning genetic analysis of PHEX, and other genes causing HR were requested.

If there was a specific diagnosis causing hypophosphatemia, such as tubulopathy or McCune Albright syndrome, clinicians were asked to specify this.

The participating centers were also asked to enter onto the system if there were any other known pathological laboratory or clinical findings.

The researchers were also asked to enter onto the system the treatment doses of phosphate and calcitiriol, and any other treatments. The yearly heights and improvement of deformities of patients who were given conventional treatment were recorded. The compliance with treatment were evaluated by asking if there were any missed planned visits and/or failure to give recommended dose of therapy by parents.

Follow-up patients were grouped as good responder (complete or significant improvement of deformities either clinically and radiologically) or bad responder (no improvement or worsening of deformities).

The follow-up form also included complications [nephrocalcinosis (NC), hyperparathyroidism, hypertension, dental abscess, cranial synostosis, entesopathy etc.] which developed during the follow-up. Entering additional information not included in the questionnaire form was optional.

### Statistical Analysis

Statistical analyses were performed by using SPSS for Windows, version 22.0, statistical software (IBM Inc., Chicago, Ill., USA). Frequencies and percentages represented the descriptive statistics for categorical variables, and mean ± SD values, median (minimum-maximum), when required, were used for continuous variables. Student’s t-test, chi-square, Fisher exact tests, repeated measures of ANOVA for normally distributed continuous variables and Friedman ANOVA as nonparametric test were used. *Post hoc* multiple comparison test was also used.

Statistically significance was regarded as p<0.05.

## Results

From 24 centers, data on 166 patients of whom 98 (59%) were females, 68 males and 18 (10.8%) pubertal and 148 prepubertal, were included in the study. The mean age of diagnosis and was 5.1±3.7 years. The mean age at beginning of symptoms and start of walking were 1.89±1.96 years and 15.5±3.82 months respectively (Table 1). Almost half of patients (n=80, 48.2%) had a history of at least one affected family member.

### Clinical and Laboratory Characteristics on Admission

The mean height SDS was -2.43±1.35. The most frequent reported clinical findings were lower limb deformities including genu varum 80.1% (n=133), genu valgum 7.8% (n=13). Bone pain 16.8% (n=28), widening of wrist 31% (n=51), rachitic rosary/thoracal abnomalities 8.4% (n=14) and frontal bossing 7.2% (n=12) were also reported. Late walking, lordosis, and congenital hip dysplasia were among the reported findings. Seven (4.2%) asymptomatic patients were detected due to a positive family history. No craniosynostosis was reported in our patient cohort.

Laboratory features were consistent with HR with hypophosphatemia, normocalcemia, high ALP, normal/normal- high PTH, and low TPR (Table 1). Mean 25-OH Vitamin D levels were 35.97±15.61 ng/mL (n=139). Low 25-OH Vitamin D levels (<20 ng/dL) were detected in 27 patients. High dose vitamin D ingestion was also reported in 16 cases due to misdiagnosis of nutritional rickets before the admission to a pediatric endocrinology clinic.

### Etiology of HR

Seven patients in our cohort had specific diagnosis from additional clinical and laboratory findings including cystinosis in three, tyrosinemia in two, Dent diseases with *CLCN5* mutation in one and McCune Albright syndrome in one patient.

**Figure 1:**
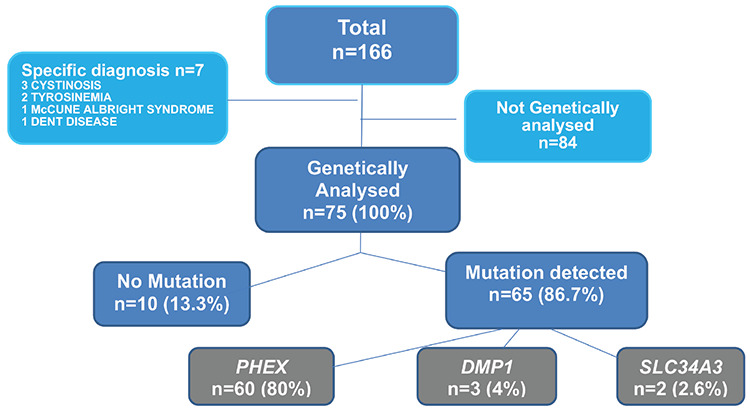
The results of genetic analysis of patients

Genetic analysis had been performed in 75 of 159 (47.2%) patients, and 65 of them showed a genetic mutation: *PHEX* mutation in 60 (80%); *DMP1* mutation in three (4%); *SLC34A3* mutation in two (2.6%) and no mutation was detected in 10 patients who were screened for *PHEX* gene by sequencing (Figure 1).

### Treatment and Follow-up

Almost all patients were treated with oral phosphate and vitamin D (calcitriol) supplements. Patients with *SLC34A3* mutation were treated solely with phosphate replacement. The mean follow-up period of the patients was 6.67±2.3 years. The first three years treatment response was evaluated for 91 patients, who had all completed at least three full years follow-up. Although an increasing trend in serum phosphate and PTH levels were seen, no statistical significant differences from initial to 1^st^, 2^nd^ and 3^rd^ year of follow-up was detected (p=0.563 and p=0.796 for serum phosphate and PTH, respectively). A decrease in ALP and ALP SD was evident (Table 2). The height SDSs were -2.38, -2.77, -2.72, -2.47 at initial, 1^st^, 2^nd^ and 3^rd^ year of treatment, respectively (p=0.570).

On follow-up, 36% of 159 HR patients without specific etiology (such as cystinosis) showed complete or significant improvement in leg deformities. Improvement in leg deformities was evaluated clinically and radiologically by each center. The patients with leg deformity improvements were compared with those who did not and both had similar ages at the time of diagnosis (4.39 vs 5.3 years, p=0.12). However, good responders had better height SDS (-2.07 vs -2.61, p=0.039), worse TPR (70% vs 77%, p=0.046), and worse ALP SDS at presentation (p=0.03). When the following years were evaluated, both groups had similar phosphate levels at presentation with better levels in the 1st and 2nd years of treatment, and even the treatment doses of phosphate were similar. However, significantly higher calcitriol treatment doses in the 1^st^ and 3^rd^ years were found in the improved group (Table 3, Table 4).

### Complications During Follow-up

When the complications of treatment were evaluated, 27 out of 159 patients (17%) developed NC on follow-up. The patients who developed NC showed no difference in biochemical characteristics at presentation and follow-up, however, their PTH levels at the 3^rd^ year were higher (145 vs. 78 pg/mL, p=0.002), and they had higher treatment dose of both phosphate and calcitriol (p<0.05) (Table 5, Table 6).

Additionally, osteotomy was required in 15 subjects (9.4%), while dental abscess occurred in 14 (8.4%) subjects, parathyroid hyperplasia developed in four subjects (2%), hypertension in one subject (<1%), and depression in one subject (<1%) among 159 cases.

Growth hormone (GH) therapy was given to 10 patients. Although duration and dose of treatment were variable, the patients treated with GH in addition to conventional therapy had similar height SDS before and after GH treatment (-3.47 and -3.3, respectively, with delta height SDS of 0.173).

## Discussion

### Characteristics at Presentation

This is the first description of a large national series of HR patient with clinical, laboratory, follow-up and etiological characteristics from Turkey. There was a delay of more than three years between the mean age of diagnosis (5.1 years) and the mean age of onset of symptoms (1.89 years). In a Norwegian series, the age of diagnosis was 2.1 years (10), also three years earlier than diagnosis in our cohort. Early diagnosis is very important for treatment response and healing especially. If treatment is started <1 year of age, it has been reported that height SDS outcome is improved (10,11).

Among the clinical findings, short stature and lower limb deformities were the most striking features. In our cohort the mean height SDS was -2.41 at presentation. Disproportionate short stature is a definitive features of HR and is primarily related to reduced growth of long bones and the limb deformities (2,11). Wide variability of height among individuals with HR has been reported and adults with XLH have a significantly reduced final height of up to 20 cm (-1.9 height SDS) (12). Almost half of our cases (80/166) had at least one individual in family with a pre-existing diagnosis of HR diagnosis but despite this positive family history, diagnosis was comparatively late in our series.

At diagnosis, one patient was diagnosed as McCune Albright syndrome, two patients with *PHEX* mutation, and two asymptomatic patients were screened because of affected siblings have normal TPR. In addition three patients had normal ALP levels, despite hypophosphatemia and low TPR on admission. Patients characteristics, mistake in sampling, or a variety of methodological problems may have led to the normal laboratory results in these patients. During follow-up all of these patients had a revised diagnosis of HR made.

Before diagnosis most of the cases were given therapeutic doses of vitamin D, on the assumption that they had calciopenic rickets. So the percentage of vitamin D deficiency (below 20 ng/dL) was correspondingly low at 19.4% (27/139) in our series at the time of diagnosis. Serum PTH levels were very helpful for diagnosis of HR. While calciopenic rickets is associated with increased PTH concentrations, our patients with HR had normal or upper normal levels of PTH.

### Etiology of HR

Seven cases in this HR cohort had a specific diagnosis of tubulopathy (cystinosis, Dent disease, tyrosinemia) or McCune Albright syndrome. Genetic analysis was performed in 75 of 159 cases. Results showed that the most frequent reason etiology of HR was XLH and 80% of patient had a *PHEX* mutation. Interestingly, this frequency was similar to other reported series in the literature in which disease-causing genetic variants were identified (3,7,8,13). We were expecting a higher frequency of autosomal recessive forms of HR in our population due to high consanguineous marriage rates. Thus our findings show that *PHEX* mutation is the most prominent form of HR regardless of the population and consanguinity rates. PHEX protein is a member of the neutral endopeptidase family of zinc metalloproteinases and predominantly expressed in osteocytes and osteoblasts (3,5). It ameliorates the inhibitory effect of matrix extracellular phosphoglycoprotein (MEPE) proteins on bone mineralization. PHEX forms a trimeric complex with DMP1 and α5β3-integrin. The resulting complex restricts FGF23 expression. While MEPE derived “acidic serine and aspartate rich motif” (ASARM) peptides inhibit the trimeric complex. So inactivating *PHEX* mutations lead to an increase both in ASARM peptides, and serum FGF23 levels (3,14). All of these caused FGF23 related phosphaturia, hypophosphatemia, short stature, and bone deformities as in our cases.

Loss of function mutation in *PHEX* causes X-linked dominant HR. Males to female ratio has previously been reported as equal (5) although in our series it was 21/32 (0.65), male to female.

In addition we found autosomal recessive HR type 1 which was detected in three cases from one family, all of whom had a *DMP1* mutation. Clinical and laboratory finding of these cases have been reported previously (15) and were similar to those seen in patients with XLH. *DMP1* mutation results in increased FGF23 production and clinical, laboratory and radiological findings could be expected to resembling those in XLH (3).

Two cases had *SLC34A3* mutations that lead to hereditary HR with hypercalciuria. This disease is characterized by hypophosphatemia and rickets, due to renal phosphorus wasting (3,8). As serum 1.25(OH)2D is high and FGF23 and PTH are reduced in such cases, secondary hypercalcemia and medullary calcinosis together with urolithiasis may be expected (3,8).

In 10 cases in which *PHEX* mutation were negative, *FGF23* had also been analyzed in six although no variants were found. Other mutations leading HR and deletions and duplication which cannot be detected by Sanger sequencing should be considered in cases with negative *PHEX* mutations.

### Treatment Results

The conventional treatment for HR includes active vitamin D analogues and phosphate supplementations. The recommended doses of calcitirol is 0.5-1.5 mcg/day or 20-30 ng/kg/day, and for phosphorus this is 20-60 mg/kg/day (16,17). In our series the doses of calcitriol and phosphorus were appropriate for recommendations but compliance of patients with treatment was a problem. This is almost always poor, due to the frequent daily dosing of drugs (4-6 times for phosphate and 1-2 times for calcitriol) and the bitter taste of the phosphate solutions. In addition the optimal dose of treatment can vary from patient to patient and higher doses will be needed during rapid growth phases and at initiation of treatment (15).

After the 3^rd^ year of conventional treatment, patients showed no improvement in height SDS. This was dissapointing but may be partly related to the late diagnosis of our patients. It is known that 25-40% of the patients with HR who are closely followed-up and compliant with treatment still have growth retardation (4). Almost 2/3 of our HR cases did not show good response to conventional treatment. Although earlier diagnosis and immediate treatment can be important prognostic factor for height improvement, we could not find any statistical differences between the good responder and bad responder groups when we stratified for age of diagnosis. The most striking feature was better height SDS in good responders compared with bad responders at presentation. This finding may indicate that bad responder were more severely affected than good responder in respect to bone pathology. Additionally, similar serum Ca and PTH levels were found in the good responder and bad responder groups. Good responders had higher serum phosphate levels despite having similar doses of phosphate replacement and higher calcitriol doses were given during follow-up.

This study has shown that conventional treatment could not lead to an additional growth promoting effect in all HR patients, but this treatment seems to stop further deterioration of growth. The effects of conventional therapy on growth have been reported previously, usually in a small case series. In a study, 13 cases showed height increment from -1.58 to -1.25 after two years of conventional therapy. In untreated historical controls (n=16) height SDS was reported as -2.02±1.30 (18). In another study, 36 cases treated with vitamin D and phosphate replacement showed improvement in linear growth, as height SDS increased from -2.89 to -1.98 (19). 

In general, bone deformities and abnormal growth of skeleton is not adequately treatable despite early and optimal doses of phosphorus and calcitriol and the lack of success with therapy for HR has led to the search for other treatment options. GH treatment is one of the therapies that has been tried in HR patients. In our series, recombinant GH (rGH) therapy was given to 10 patients. However, these patients showed similar height increment to patients receiving only conventional treatment. rGH treatment in HR has usually been reported in limited pilot studies, although these have suggested a beneficial effect. While one randomized study showed significant improvement in height SDS in eight severely short XLH children treated with rGH for three years, follow-up showed that this treatment did not significantly increase adult height (20,21).

During follow-up, corrective osteotomy was required in 15 (9.4%) among the 159 subjects.

Development of progressive bone deformities in HR could lead to progressive gait disturbance, functional impairment, and severe arthritis. In such conditions surgical treatment may help improve patients quality of life (22). Each patient should be evaluated carefully for requirement for corrective surgery.

### Complications During Follow-up

The compliance of treatment is a difficult issue in HR, since phosphate needs 4-6 daily doses and calcitriol needs 1-2 daily doses. During conventional treatment of HR, several complications may occur including NC, dental abscesses, entesopathy, and craniosynostosis. Among these, NC is more frequently reported (1,2,3,4,17).

In this national cohort 17% of all hereditary hypophosphatemia patients had NC, which is a relatively low rate compared to other series, with NC being reported in 22% to 100% of XLH patients who are under conventional therapy (1,2,3,4). However, these were small series and diagnosis was earlier than in the present study. We speculate that earlier therapy may lead to an increased frequency of NC.

NC usually develops after conventional treatment of HR, and is accepted as a treatment complication. It is known that higher doses of phosphate replacement caused both to NC and hyperparathyroidism. Higher doses of phosphate replacement may increase FGF23, and phosphaturia will be increased (13). Similarly, our cases with NC were treated with higher doses of phosphate than the cases without NC. In addition to higher dose of phosphate replacement, calcitriol may lead to hypercalcemia which may be additive in the likelihood of NC occurrence. In our cases higher doses of calcitriol during initial therapy seems to have had an additional effect on NC development. The reason for high dose therapy were not recorded in all cases. However, some physicians might have belived that response to therapy was insufficient and were attempting to maintain the serum phosphate levels within the reference values. In fact, the primary aim in conventional HR therapy should be to keep serum Ca, ALP, PTH, and urinary Ca excretion in normal limits rather thn the phosphorus concentration. It is salutory that although higher doses of phosphate and calcitriol were given, possibly predisposing some patients to NC, this NC group did not have better growth outcomes than the lower dose patients who did not develop NC.

While dental abscess was present in 14 cases, other complications were infrequently reported, with parathyroid hyperplasia in four (2%), hypertension in one (<1%), and no neurological complications including craniosynostosis. Awareness of the complications of HR should be improved among medical professionals.

The aim of conventional treatment is to compensate for renal phosphate wasting and to counter 1,25OHD deficiency (4), which is commonly related to excess levels of FGF23 in HR. Recently, some strategies that manipulate FGF23 signaling have been described. Burosumab, a monoclonal antibody directed at FGF23, is one (23). In children with HR, treatment with burosumab has been reported to improve renal tubular phosphate reabsorbsion, serum phosphate levels, linear growth, and severity of rickets (24) but burosumab is expensive and long-term outcomes are not yet available.

Conventional therapy is still the first line therapy for HR. When patients are correctly treated with conventional therapy, the incidence of NC should be lower, and good proportion of patients would have a good response.

### Study Limitations

Ascertainment of all case data for the whole country was not complete and inevitably some findings were not reported. In addition, as the radiological findings were evaluated in each center, follow-up characteristics were mainly based on the healing of skeletal deformities, which was assessed subjectively by many different radiologists. Unfortunately genetic diagnosis was not made in all cases. Almost half of all cases were analysed and definitive PHEX mutation was found in 80% of genetically analysed cases but this amounted to only 36% of all cases.

## Conclusion

Age of diagnosis in HR patients was late in our series, despite some having positive family history, and the most frequent etiology was *PHEX* mutation. HR treatment and follow-up is challenging and our results showed an association between higher treatment doses and NC without any change in serum levels or growth outcomes, suggesting that higher doses lead to increased phoshaturia, probably through the stimulation FGF23. However, higher calcitriol doses appear to improve bone deformities.

## Figures and Tables

**Table 1:**
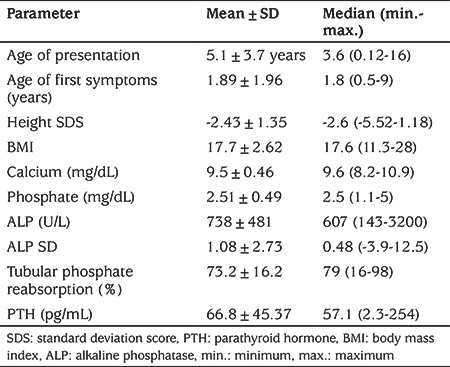
Clinical and laboratory characteristics of all cases on admission

**Table 2:**
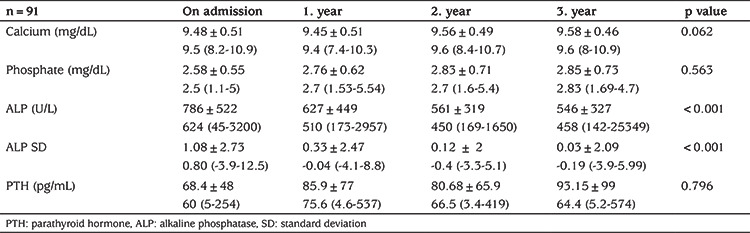
Laboratory evaluation of 91 cases during 3 years of follow-up

**Table 3:**
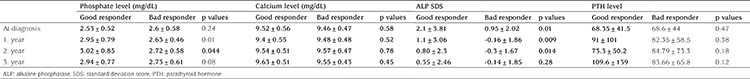
Laboratory characteristics of good responder and bad responder groups for 3 years follow-up

**Table 4:**
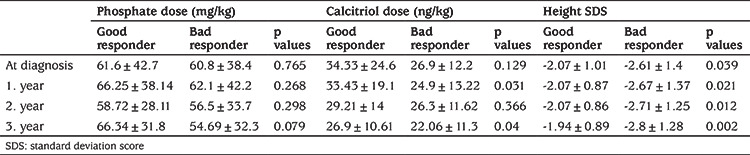
Doses of treatment and growth characteristics of good responder and bad responder groups for 3 years follow-up

**Table 5:**
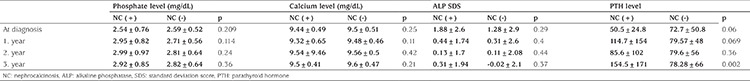
Laboratory characteristics of patients according to development of nephrocalcinosis

**Table 6:**
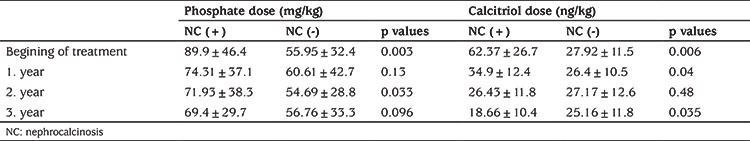
Treatment characteristics of patients according to development of nephrocalcinosis
